# Distribution of *Merlin* in eukaryotes and first report of DNA transposons in kinetoplastid protists

**DOI:** 10.1371/journal.pone.0251133

**Published:** 2021-05-06

**Authors:** Ana Luisa Kalb Lopes, Eva Kriegová, Julius Lukeš, Marco Aurélio Krieger, Adriana Ludwig

**Affiliations:** 1 Pós-Graduação em Biologia Celular e Molecular, Universidade Federal do Paraná, Curitiba, PR, Brazil; 2 Laboratório de Ciências e Tecnologias Aplicadas em Saúde (LaCTAS), Instituto Carlos Chagas, Fundação Oswaldo Cruz (Fiocruz), Curitiba, PR, Brazil; 3 Instituto de Biologia Molecular do Paraná, Curitiba, PR, Brazil; 4 Institute of Parasitology, Biology Center, Czech Academy of Sciences, České Budějovice (Budweis), Czech Republic; 5 Faculty of Sciences, University of South Bohemia, České Budějovice (Budweis), Czech Republic; University of Bari, ITALY

## Abstract

DNA transposons are defined as repeated DNA sequences that can move within the host genome through the action of transposases. The transposon superfamily *Merlin* was originally found mainly in animal genomes. Here, we describe a global distribution of the *Merlin* in animals, fungi, plants and protists, reporting for the first time their presence in Rhodophyceae, Metamonada, Discoba and Alveolata. We identified a great variety of potentially active *Merlin* families, some containing highly imperfect terminal inverted repeats and internal tandem repeats. *Merlin*-related sequences with no evidence of mobilization capacity were also observed and may be products of domestication. The evolutionary trees support that *Merlin* is likely an ancient superfamily, with early events of diversification and secondary losses, although repeated re-invasions probably occurred in some groups, which would explain its diversity and discontinuous distribution. We cannot rule out the possibility that the *Merlin* superfamily is the product of multiple horizontal transfers of related prokaryotic insertion sequences. Moreover, this is the first account of a DNA transposon in kinetoplastid flagellates, with conserved *Merlin* transposase identified in *Bodo saltans* and *Perkinsela* sp., whereas it is absent in trypanosomatids. Based on the level of conservation of the transposase and overlaps of putative open reading frames with *Merlin*, we propose that in protists it may serve as a raw material for gene emergence.

## Introduction

Transposable elements (TEs) are defined as repeated DNA sequences that can move within the host genome. TEs are not only present in both prokaryotes and eukaryotes, but they also constitute a significant fraction of numerous genomes, including those of humans [1] and plants [2, 3]. TEs produce various genetic alterations that play a central role in the structural organization and plasticity of genomes [4]. Their insertions have the potential to inactivate or alter the expression of genes or gene regulatory elements. By ectopic recombination, TEs may trigger chromosomal rearrangements and contribute to mutagenesis [5–7]. Moreover, TEs are rich in coding and regulatory sequences that can be co-opted by the host to develop novel cellular functions in a process called domestication or exaptation [8–10].

TEs exhibit a broad diversity in their structure and transposition mechanisms. A unified classification system for eukaryotic TEs was proposed, establishing two classes according to their transposition mechanisms, structures, and sequence similarity. Class I elements or retrotransposons move by a copy-paste mechanism that involves the reverse transcription of an RNA intermediate and insertion of its cDNA copy at a new site in the genome. Class II elements, or DNA transposons, move through a DNA intermediate [6, 11]. The classical DNA transposons consist of a transposase gene that is flanked by two terminal inverted repeats (TIRs) forming the so-called TIR order [6]. During transposition, the transposase enzyme recognizes the TIRs and performs the excision of the transposon by double-strand DNA breaks, which is followed by the insertion into a new genomic location. Upon insertion, the target DNA site is duplicated, resulting in target site duplications (TSDs) [12]. The first 9 recognized TIR superfamilies [6] are distinguished by their sequences and the TSD size (*Tc1/mariner*, *PIF/Harbinger*, *hAT*, *Mutator*, *Merlin*, *Transib*, *P*, *piggyBac* and *CACTA*). Currently, in Repbase [13], a database of eukaryotic TEs and repetitive sequences, 18 superfamilies encoding a D-D-D/E-type transposase are distinguished.

The *Merlin* superfamily was first described by Feschotte in 2004 [14] as a group of elements detected by computational analysis in a wide range of genomes, which share common structural features and sequence motifs. These elements possess TIRs that range in length from 24 to 462 base pairs (bp) with conserved terminal 5’-GG-3’, and are flanked by 8-bp or 9-bp TSDs. *Merlin* elements were found to be related to the IS*1016* group of bacterial insertion sequence (IS), sharing sequence similarity in the C-terminal halves of the proteins and the TSD size [14]. IS*1016* together with IS*Pna2*, IS*H4*, IS*1595*, IS*Sod11*, IS*Nwi* and IS*Nha5* constitute a major group so-called the IS*1595* family [15]. Members of this IS family are usually flanked by 8-bp TSDs, have a single transposase gene, and, except for the IS*1016* group, all others have an N-terminal zinc finger domain [15] named Zn_Tnp_IS1595. They also have a conserved C-terminal domain containing the DDE catalytic motif shared with the eukaryotic *Merlin* transposons [14, 15] named DDE_Tnp_IS1595. All *Merlin* and IS*1016* elements described so far lack the Zn_Tnp_IS1595 domain.

Elements from superfamily *Merlin* were identified in the genome of several animals, including nematodes, flatworms, mosquitos, ascidians, zebrafish, frogs, and its relics are also present in humans [14]. Outside animals, the *Merlin* superfamily was described from the oomycete *Phytophthora sojae* (Stramenopila), the microsporidian *Nosema bombycis* (Microspora) [14], fungi and embryophytes [16]. Moreover, in the microsporidian *Anncaliia algerae*, *Merlin* represents the most abundant sequence element and seems to be involved in horizontal transfer events [17].

Within the last decade, the number of newly sequenced genomes is accelerating, providing data for an increased rate of identification of TEs. Here, we have performed bioinformatic analyses and updated our knowledge of the distribution of *Merlin* across eukaryotes, documenting for the first time its presence in Alveolata, Rhodophyceae, Metamonada and Discoba. This work also comprises the first record of DNA transposons in the well-studied kinetoplastid protists.

## Materials and methods

### Searches for *Merlin* sequences

It is possible to observe that canonical elements from IS*1595* family (data not shown) and eukaryotic *Merlin* elements (S1 Table) have the DDE_Tnp_IS1595 domain (NCBI CDD accession: cl01481), which is on average 130 amino acids (aa) long, being a diagnostic feature.

*Merlin* sequences deposited in the Repbase database (http://www.girinst.org/repbase/) [13] were analyzed for the conservation of the DDE_Tnp_IS1595 domain using the NCBI CD-search tool [18]. Among them, Merlin1_SM from *Schmidtea mediterranea* was used in the initial blastp searches from NCBI server [19] against distinct eukaryotic groups. Sequences presenting a hit with an e-value lower than 1e-04 were retrieved and evaluated for the presence of the conserved domain. The region corresponding to the DDE_Tnp_IS1595 domain was extracted from all sequences retrieved from Repbase and blastp searches (around 300 sequences). Next, these sequences were used as query sequences in the second round of blastp searches (e-value cutoff of 1e-03) against each of the higher eukaryotic ranks, according to the classification of Adl et al. (2019). All retrieved sequences were analyzed in the CD-search for the presence of expected and other domains. To expand searches to taxa that did not produce significant hits in the blastp but have their genome sequence available, we performed online or local tblastn searches using the same e-value cutoff. Possible significant hits were confirmed by extracting the sequences from the genomes (using an *in-house* Python code) and searching for open reading frames (ORFs) (using NCBI ORFfinder tool) and the DDE_Tnp_IS1595 domain by CD-search. Most blast searches were completed until November 2019.

To check for the possibility of bacterial sequence contamination, the retrieved proteins were used as a query on blastp against the nr NCBI database. Hits with more than 80% identity with a bacterial sequence were considered possible contaminations.

### Structural analysis of *Merlin* in Rhodophyceae, Metamonada, Discoba and Alveolata

Since significant hits were found by blastp in several alveolates, all genomes available for this group were analyzed by tblastn to recover possible complete elements. For the analysis of significant tblastn hits from Rhodophyceae, Metamonada, Discoba and Alveolata, we prioritized the curated analysis to genomes where copies are more conserved and located in long contigs/scaffolds. Sequences retrieved from the same species were aligned with MAFFT [20], and the alignments were inspected to identify the limits of the copies. To verify the presence of the TIRs and TSDs, most conserved copies were analyzed by blastn with the parameters “align two or more sequences” and “somewhat similar sequences (blastn)” using the same sequence as a query and subject, and by visual inspection of alignments. Similarity with the eukaryotic TEs was checked using CENSOR tool from Repbase [21]. To evaluate divergence among copies in some species, protein and nucleotide sequences were aligned using MAFFT, and Mega X [22] was used to cluster the sequences using neighbor-joining (p-distance, 1000 replicate bootstrap) and to access the pairwise p-distance among them. The online Tandem Repeats Finder program [23] was used to help identify tandem repeats inside the *Merlin* copies. The secondary structure of *Merlin* from the kinetoplastids *Bodo saltans* and *Perkinsela* sp. was analyzed by Phyre2 [24]. All sequences analyzed are available in S1–S3 Appendix.

### PCR confirmation of *Merlin* in *Perkinsela* sp

The DNA of *Perkinsela* sp. (strain CCAP1560/4) was submitted to gradient PCR analysis. Primers were designed to anneal inside the transposon and the up- or downstream genes. Single-copy genes and those that are present across the kinetoplastid flagellates were chosen. More information about the primers and the amplification conditions is available in S1 File.

### Evolutionary analyses

The Transposase DDE_Tnp_IS1595 domain was isolated from identified sequences and filtered by size (>115 aa). Sequences were then filtered by identity (70%) using CD-HIT [25] to reduce the number of sequences and produce a less complex matrix. Three sequences from each group of IS*1595* family, as classified in IS Finder (https://www-is.biotoul.fr/index.php), were added to the matrix (S2 Table). Sequences were aligned using PROMALS3D [26]. The alignment was trimmed using trimAl 1.4.1 [27] (0.4 of GAP threshold; 0.0 of similarity threshold). Additional filtering was done to eliminate some short sequences (<110 aa) from the final matrix. The evolutionary model LG+G was indicated by the test implemented in Mega X [22]. An additional alignment matrix was constructed including only the copies that have been curated by us and the ones from Repbase together with the IS*1595* group sequences with no size filtering. For this matrix, the evolutionary model WAG+G was indicated. The trees were inferred by Bayesian Analysis (BA) in MrBayes 3.2.6 [28] with the indicated model and were run in the CIPRES gateway [29]. The Markov Chain Monte Carlo (MCMC) of the BA was run for at least 10,000,000 generations, sampling trees every 1,000 generations, and burning 25% of the initial results. Additionally, a maximum likelihood (ML) analysis with the indicated model was performed under the rapid bootstrap algorithm using RAxML-HPC BlackBox [30] implemented on CIPRES with automatic determination of bootstrap replicates.

## Results and discussion

### *Merlin* is widely distributed in eukaryotes

As new genomes from a broad range of taxa become available at an ever-increasing pace, it is possible to expand our knowledge about the distribution and evolutionary history of TEs. Here, we describe a broad analysis of the distribution of *Merlin* in eukaryotes. For this, we first performed a search for *Merlin* deposited in Repbase and identified 70 sequences, of which 32 displayed the expected DDE_Tnp_IS1595 domain and the DDE motif, an essential part of the catalytic site of transposases [31]. In order to carry out a global search for these elements, we performed blastp searches in two rounds, using the sequences retrieved in the first search as multi-queries in a second round of blastp in specific taxonomic groups, increasing the chances to find similar sequences. The sequences were considered positive hits for *Merlin* only if the DDE_Tnp_IS1595 domain was found with no evidence of bacterial contamination.

**Fig 1:**
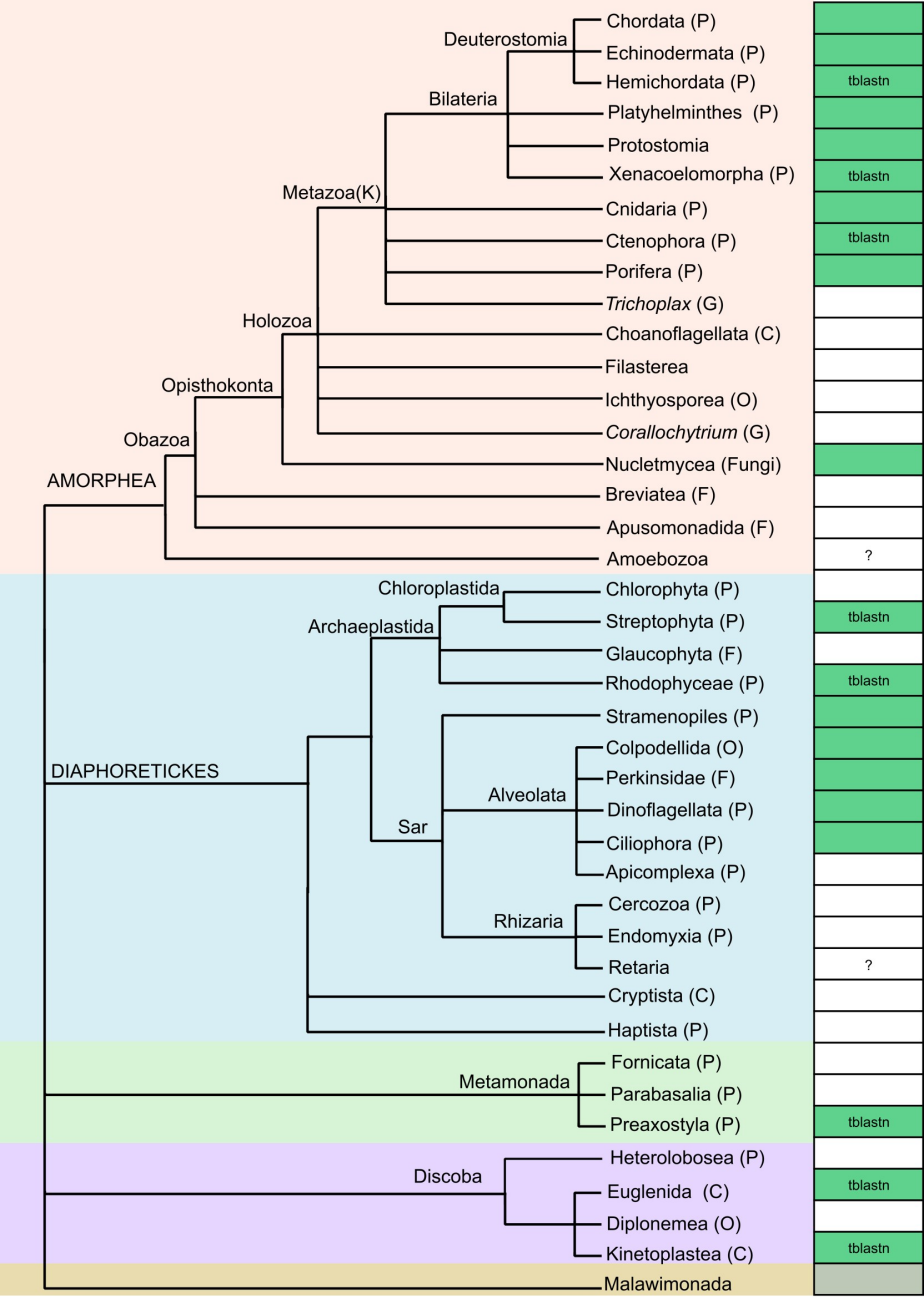
Phylogenetic distribution of the *Merlin* elements in eukaryotes. The cladogram was drawn based on [32], and the subdivisions of Bilateria followed the NCBI Taxonomy. Green boxes indicate the presence of *Merlin* in at least one species per each taxonomic group and those analyzed by tblastn were indicated; gray boxes indicate that the group has no genome sequence available; white boxes indicate that no *Merlin* sequence was found;? indicates that the presence of *Merlin* remains unclear. G = genus; F = family; O = order; C = class; P = phylum; K = kingdom. Some taxa that have no genome available were omitted from the three (Chaetognatha, Gnathostomulida, Syssomonas from Holozoa; Colponemidia, Acavomonas from Alveolata; Jacobida, *Tsukubamonas* from Discoba: Symbiontida from Euglenozoa).

Our results show that *Merlin* is present in a wide range of animals, fungi, plants and protists and for the first time, it was found in Alveolata, Rhodophyceae, Metamonada and Discoba species (Fig 1; S3 Table). For taxonomic assignments, we have used the recently revised classification of eukaryotes [32].

In Bilateria, few copies per species were found (Chordata: 90 sequences in 31 species; Echinodermata: 8 sequences in 2 species; Platyhelminthes: 58 sequences in 9 species; Protostomia: 1269 sequences in 126 species) (S1 Appendix). However, in a subset of non-closely related species, *Merlin* was more abundant, such as in the flatworm *Clonorchis sinensis* (24 sequences) and several protostomians, namely the octopid *Octopus bimaculoides* (191 sequences), the whipworm *Trichuris suis* (147 sequences), the spider *Araneus ventricosus* (112 sequences), the scorpion *Centruroides sculpturatus* (48 sequences) and few others.

In Cnidaria, 10 species showed significant hits varying from 5 to 23 sequences, except for the myxosporean *Thelohanellus kitauei*, where 185 significant hits were found (S1 Appendix). In fact, Yang et al. found 179 transposon sequences with the DDE_Tnp_IS1595 domain in the genome of this parasite, although they did not classify them as *Merlin* [33]. In Porifera, only 3 sequences were found in *Amphimedon queenslandica* (S1 Appendix).

In Nucletmycea, a group that includes fungi, 888 sequences were found in 28 genomes (S1 Appendix). A high number of sequences is present in two distinct strains of *A*. *algerae* (338 and 281 sequences) and other microsporidian species, namely *Nosema ceranae* (40 sequences), *Pseudoloma neurophilia* (38 sequences) and *Hepatospora eriocheir* (32 sequences). Evidence of a relatively recent spread of *Merlin* was reported in some microsporidian species [34]. Furthermore, *Merlin* was found in Mucoromycota (*Rhizopus delemar*: 57 sequences; *Lichtheimia corymbifera*: 14 sequences; *Phycomyces blakesleeanus*: 11 sequences; *R*. *microspores*: 10 sequences, and others), a clade consisting of mycorrhizal fungi, root endophytes, and decomposers of plant material [35]. The queried elements were also identified in single representatives of the basal fungal phyla Cryptomycota (4 sequences in *Rozella allomycis*, also found by [36]) and Zoopagomycota (a single sequence in *Conidiobolus coronatus*).

The presence of *Merlin* in stramenopiles was previously reported [14] and here we found 128 sequences in 13 genomes from the genera *Phytophthora* and *Aphanomyces* (S1 Appendix). On the other hand, we provide the first report of *Merlin* in several alveolates mainly from Ciliophora, Dinophyceae and Perkinsozoa, and we explored its structure in more detail (see below).

By tblastn, possible *Merlin* elements were also found in 2 hemichordate genomes (S2 Appendix). *Saccoglossus kowalevskii* (GCA_000003605.1) showed around 30 hits, most of which represent remnants of *Merlin*, and 2 preserved transposase ORFs were found. In contrast, hundreds of hits were found in *Ptychodera flava* (GCA_001465055.1) along with at least 8 preserved transposase genes. Moreover, hundreds of significant hits were found in 2 available Xenacoelomorpha genomes, namely *Hofstenia miami* (GCA_900660155.1 and GCA_004352715.1) with at least 10 preserved transposase ORFs. Some hits were also found in 3 analyzed ctenophores genomes: *Mnemiopsis leidyi* (GCA_000226015.1; only remnants of ORFs), *Pleurobrachia bachei* (GCA_000695325.1; 4 preserved ORFs) and *Beroe ovata* (GCA_900239995.1; 6 preserved ORFs).

In Rhodophyceae, Metamonada and Discoba, we also found significant hits by tblastn, corresponding to the first report of *Merlin* superfamily in these taxa and we performed a curated analysis of copies (see below).

Some hits were also found in the genomes of 3 green algae (Chlorophyta) from 97 genomes analyzed, namely *Dunaliella* sp. (GCA_004335775.1), *Chloromonas* sp. (GCA_004335635.1) and *Ulva prolifera* (GCA_004138255.1) (S2 Appendix). Since in *U*. *prolifera*, the transposase and flanking regions have high similarity to bacterial sequences (around 90%), contamination is strongly suggested. Although sequences from the other two species share only around 40% identity with bacterial sequences, they seem to be contaminations as well, since near genes also have these similarities and the contaminants contigs are nearly identical in both genomes.

From 45 analyzed amoebozoan genomes, hits were found in *Acanthamoeba mauritaniensis* (GCA_000826465.1), *Physarum polycephalum* (GCA_000413255.3) and *Synstelium polycarpum* (GCA_900092255.1) (S2 Appendix). Transposase and near genes identified in *A*. *mauritaniensis* and *P*. *polycephalum* are 90–100% identical with bacterial sequences. Hits for *S*. *polycarpum* are from truncated ORFs located in 4 very short contigs (488–941 bp) that do not show high similarity with bacterial sequences. However, since these contigs are not mapped on the genome, it is hard to judge whether these are remnants of *Merlin* or contaminations with eukaryotic reads. Thus, we do not have clear evidence for the presence of *Merlin* in this group.

In Rhizaria, significant hits were found in only 2 genomes from 60 analyzed ones of 11 species (S2 Appendix). In *Globobulimina* sp. (GCA_003354225.1) *Merlin* hits are from truncated ORFs located in 2 very short contigs with no evidence of bacterial contamination. Similar to *S*. *polycarpum*, it is not clear if these sequences are part of the genomes. In *Reticulomyxa filosa* (GCA_000512085.1) some significant hits containing truncated ORFs and 2 complete ORFs were found. A blastp of the transposases and neighboring genes revealed 40–60% identity to bacterial sequences. Despite a relatively low similarity, this might be contamination, since the contigs are not assembled in the genome. A hypothesis of horizontal transfer of bacterial fragments is unlikely because the flanking genes are distinct among contigs, and consequently, several independent events have to be implied. Glöckner et al (2014) have identified and removed some contigs in the *R*. *filosa* assembly that were derived from bacteria [37], but the contigs we identified are possibly from an unknown bacterial source that escaped this filtering. Thus, the presence of *Merlin* sequences in Rhizaria remains unclear.

Using blastp, we were unable to identify significant hits in plants (Streptophyta), although one *Merlin* element was previously identified in the spikemoss *Selaginella moellendorffi*, and is deposited in the Repbase database (Merlin-1_Smo) [13, 16]. We have also analyzed almost 700 genomes using tblastn, yet most hits appear to be contaminations. For example, one significant hit was found in a short contig of the *Lactuca sativa* genome (GCA_900243165.1) that has 99.91% identity to a genomic fragment of the bacterium *Proteiniphilum saccharofermentans*. Nevertheless, for a few species, there was no evidence of sequence contamination and *Merlin* is indeed present in their genomes, such as in *S*. *moellendorffii* (GCA_000143415.2), *S*. *kraussiana* (GCA_001021135.1) and *S*. *tamariscina* (GCA_003024785.1). However, functionally active copies are absent. The canonical Merlin-1_Smo and the copies found in the genomes have truncated DDE_Tnp_IS1595 domain. Since the genus *Selaginella* belongs to a basal lineage (class Lycopodiopsida) [38], it is plausible to assume that *Merlin* was lost in more derived plant lineages. This notion is supported by our finding of degenerate copies in *Rhodamnia argentea* (GCA_900635035.1) from the derived clade Euphyllophyta.

In some groups, for which whole genomes are available, namely *Trichoplax* (2 genomes), Choanoflagellata (2 genomes), Filasterea (1 genome), Ichthyosporea (9 genomes), *Corallochytrium* (1 genome), Breviatea (1 genome), Apusomonadidae (1 genome), Glaucophyta (1 genome), Cryptista (6 genomes), and Haptista (3 genomes), no significant hits for *Merlin* were found neither by blastp nor by tblastn.

It is important to notice that using blastp searches, we were looking for annotated proteins containing at least a partial DDE_Tnp_IS1595 domain and consequently, more degenerate copies were not identified. The analysis of a CD-search indicates that for some proteins the DDE_Tnp_IS1595 domain is incomplete (S1 and S2 Appendix) and these are possibly inactive transposases. Also, in several proteins, we observed additional domains that are not expected to be found in transposases such as the EGT51828.1 protein from *Caenorhabditis brenneri* that has a DDE_Tnp_IS1595 followed by a WD40 domain. These proteins could be a result of degeneration or yet artifact of genomic misassembly but are also are good candidates for functional chimeras. Recently, Cosby et al. identified several examples of host-transposase fusion genes as a recurrent path for the emergence of transcription factors [39]. Interestingly, the Zn_Tnp_IS1595 domain was predicted in the N-terminal portion of a few proteins. For example, this domain is found in two proteins from *C*. *briggsae* (XP_002642078.1 and XP_002638395.1) with no indication of bacterial sequence contamination, questioning whether all eukaryotic *Merlin* are derived from IS*1016* that lacks this domain.

In general, we observed a wider distribution of *Merlin* than previously reported. Although we did not explore the presence/absence in lower taxonomic ranks, *Merlin* is clearly absent from a number of taxa, which may be a result of multiple independent losses and/or horizontal transfer. Extensive sequencing of the underrepresented taxa should help to distinguish between these two possibilities. The picture is further confused by contaminations that represent a common occurrence in large-scale sequencing and usually derive from microbiome present in the analyzed tissues or from the environment [40–43].

### First report of *Merlin* in Rhodophyceae, Metamonada, Discoba and Alveolata

**Fig 2:**
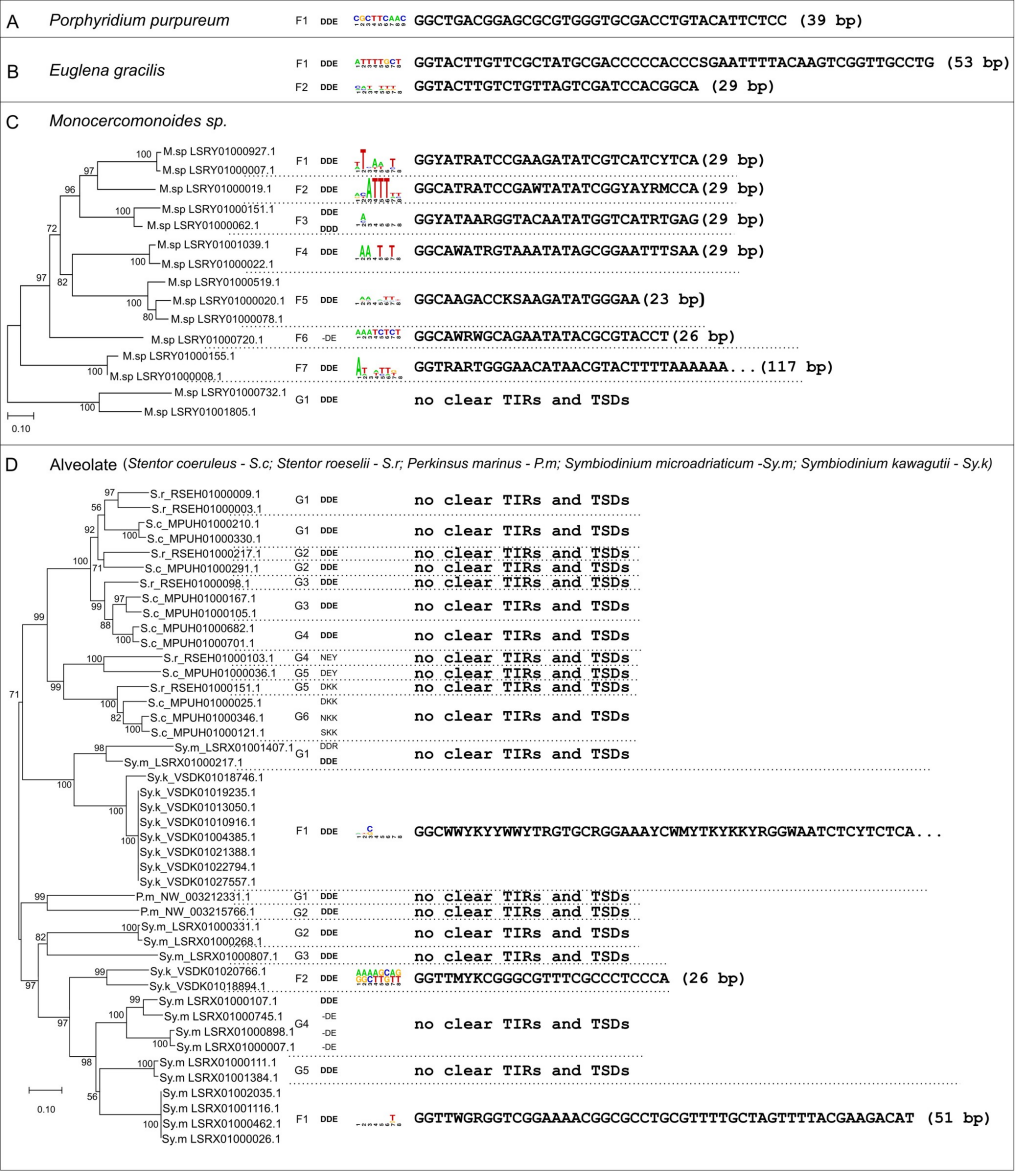
Main characteristics of *Merlin* sequences that were identified in this work. Similar *Merlin* copies within the same species sharing TIR sequences were grouped into families and identified by the letter F followed by a number. Groups of sequences within the same species with no TIRs and TSDs were divided according to the nucleotide divergence and identified by the letter G followed by a number. The residues aligned in the positions of the DDE motif are shown, and its conservation is highlighted in bold. TSD logos are shown and represent the nucleotide usage at each position and the y-axis ranges from a bit score of zero to two. TIR sequences are also shown and represented by both the 5’ TIR and the reverse complement of the 3’ TIR. Sequences are majority-rule consensus derived from the alignment of multiple copies of each family or individual copies in some cases and the mismatches between the two TIRs are shown as degenerate bases (R = A or G, Y = C or T, S = G or C, W = A or T, K = G or T, and M = A or C). A) *Merlin* family from *P*. *purpureum* has conserved DDE motif, TIRs of 39 bp and the 9-bp TSDs logo is a frequency plot based on one conserved copy. B) *Merlin* families (F1 and F2) from *E*. *gracilis* carry the conserved DDE motif, 8-bp TSDs and almost perfect TIRs. TSD logo from F1 is a frequency plot based on one preserved copy. C) Neighbor-joining tree of *Merlin* transposase proteins found in *Monocercomonoides* sp. showing at least 7 families (F1-F7) that present different TIRs and no clear TSDs consensus. D) Neighbor-joining tree of *Merlin*-related proteins found in Alveolata (S.r–*Stentor roeselii*; S.c–*S*.*coeruleus*; P. p—*Porphyridium purpureum*; Sy.m–*Symbiodinium microadriaticum*; Sy.k–*Symbiodinium kawagutii;* P.m–*Perkinsus marinus*) based only on the conserved DDE_Tnp_IS1595 domain. However, the DDE motif is not conserved in all sequences and TIRs and TSDs were identified in only a few of them. TSD logo from F2 is a frequency plot based on two conserved copies. The limit of the TIRs from F1 is not clear.

**Fig 3:**
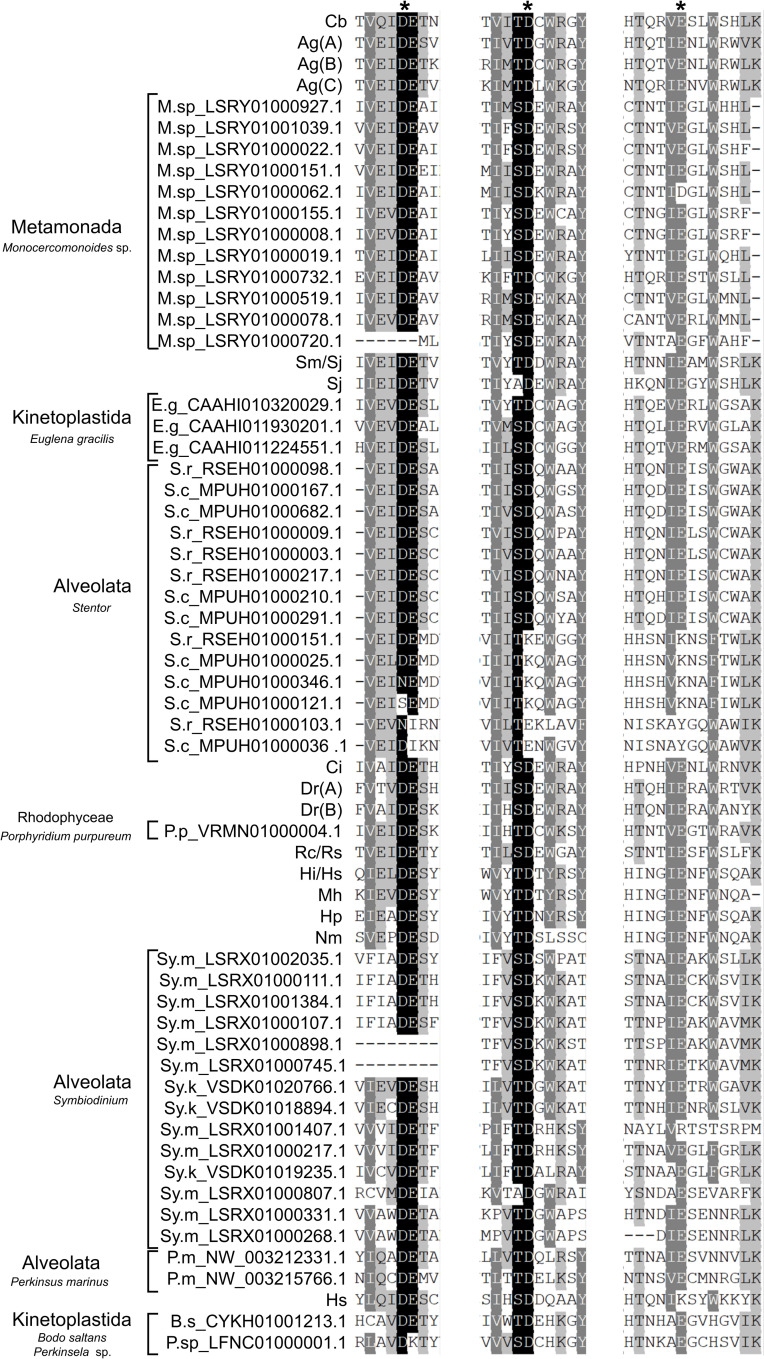
Alignment of the DDE catalytic motif region of *Merlin* families. The three conserved blocks of residues surrounding the DDE motif identified by Feschotte [14] are shown. The number of residues between blocks 1 and 2 varied from around 50 to 70 aa, except for the sequences Sy.m_LSRX01000331.1, Sy.m_LSRX01000268.1 and Sy.m_LSRX01000807.1 that present a larger region (around 110 aa). The DDE motif positions are highlighted with asterisks above the alignment. Colours on the sequences denote residues conservation: black > 90%; dark grey > 80%; light gray > 60%. All *Merlin* transposase proteins identified in this work were aligned with the *Merlin* sequence from *C*. *briggsae* (CAE74230). The consensus sequences for the three blocks of other *Merlin* transposases and IS*1016* were obtained from [14] and added to the alignment (Cb–*C*. *briggsae*; Tm–*Trichuris muris*; Ag(A), Ag(B) and Ag(C)–*Anopheles gambiae;* Sm/Sj–*Schistosoma mansoni* and *S*. *japonicum*; Sj–*S*. *japonicum;* Ci–*Ciona intestinalis*; Dr(A) and Dr(B)–*Danio rerio*; Hs–*Homo sapiens*; Rc/Rs—*Rickettsia conorii* and *R*. *sibirica*, Hi/Hs–*Haemophilus influenzae* and *H*. *somnus*, Hp–*H*. *paragallinarum*, Mh–*Mannheimia haemolytica*; Nm–*Neisseria meningitides*). Sequences from this work are identified by initials (M.sp–*Monocercomonoides* sp.; E.g–*Euglena glacilis*; S.r–*Stentor roeselii*; S.c–*S*.*coeruleus*; P. p—*Porphyridium purpureum*; Sy.m–*Symbiodinium microadriaticum*; Sy.k–*Symbiodinium kawagutii;* P.m–*Perkinsus marinus*; B.s–*Bodo saltans*; P. sp–*Perkinsela* sp.) and the contig/scaffold ID. Some copies that are identical to others in these regions were omitted from the alignment (Sm_LSRX01000007.1 equal to Sm_LSRX01000898.1; M.sp_LSRY01000007.1 equal to M.sp_LSRY01000927.1; M.sp_LSRY01000020.1 equal to M.sp LSRY01000078.1; M.sp LSRY01001805.1 equal to M.sp LSRY01000732.1; Sc_MPUH01000330.1 equal to Sc_MPUH01000210.1; Sc_MPUH01000105.1 and Sc_MPUH01000701.1 equal to Sc_MPUH01000682.1; Sy.k_VSDK01018746.1, Sy.k_VSDK01013050.1, Sy.k_VSDK01010916.1, Sy.k_VSDK01004385.1, Sy.k_VSDK01021388.1, Sy.k_VSDK01022794.1 and Sy.k_VSDK01027557.1 equal to Sy.k_VSDK01019235.1; Sy.m_LSRX01001116.1, Sy.m_LSRX01000462.1, Sy.m_LSRX01000026.1 equal to Sy.m_LSRX01002035.1).

From 20 analyzed Rhodophyceae genomes, *Merlin*-related sequences were found only in *Porphyridium purpureum* (GCA_008690995.1) (S4 Table). Two copies were found sharing 99% nucleotide identity, and the alignment revealed imperfect TIRs of 39 bp, extendable with additional mismatches. One of these copies presents conserved TSDs of 9 bp that indicates recent insertion (Fig 2A, S3 Appendix). They encode proteins of 319 aa with conserved DDE_Tnp_IS1595 domain and DDE motif (S4 Table and Fig 3). CENSOR analysis indicates low similarity (33–39%) with known *Merlin* from Repbase. Only one additional remnant copy of *Merlin* was found in the genome. The presence of at least one potentially active and new copy and the absence in other red algae suggest a recent invasion of *Merlin* in this genome.

In Discoba, 95 genomes were searched (S4 Table), and significant hits were found in *Euglena gracilis* (GCA_900893395.1) and 2 kinetoplastids (see below). Most hits found in *E*. *gracilis* are in short contigs that hindered the recovery of complete copies (S3 Appendix). However, we were able to establish two different *Merlin* families based on the composition and size of TIRs and sequence divergence, both generating 8-bp TSDs (Fig 2B). They carry the expected conserved domain and present 47 to 56% similarity with *Merlin* from *S*. *mediterranea* (S4 Table); the DDE motif is conserved (Fig 3).

In Metamonada, 24 genomes from 3 phyla were investigated (S4 Table), being found only in Preaxostyla. Few remnants of the *Merlin*-related sequences are found in *Streblomastix strix* (GCA_008636045.1) with no evidence of sequence contamination. It could be a result of an old invasion not followed by a successful amplification. On the other hand, the *Monocercomonoides* sp. PA203 genome (GCA_001643675.1) contains over 100 copies of *Merlin* with preserved or broken transposase ORFs (S3 Appendix). Within the most conserved sequences, we have found at least 7 *Merlin* families (Fig 2C) with different compositions of TIRs, all generating TSDs of 8 bp. Most families have TIRs ranging from 23 to 29 bp, but in some cases, the internal borders of those TIRs were not very clear, since they could be extended considering additional mismatches. The corresponding proteins have 28 to 42% similarity with other *Merlin* elements (S4 Table) and do not seem to be contaminations. As expected for functional transposases, copies of almost all families have the conserved DDE motif (Fig 3). We suggest that the high number of *Merlin* families in this species is the result of an ancient invasion followed by diversification. We found different levels of degeneration, with the transposase pseudogenization occurring in several copies that still contain nearly conserved TIRs and TSDs.

From 340 analyzed alveolate genomes, sequences homologous to *Merlin* were initially found in 7 species by blastp and subsequently in 33 genomes by tblastn. Significant hits were found in ciliates (24 from 33 genomes), dinoflagellates (5 from 7 genomes), Perkinsidae (3 from 8 genomes), and Coolpodellida (1 genome from 2 genomes). In 290 apicomplexans genomes tested, a taxon known to be devoid of TEs [44], only possible contaminants were found in two genomes (S4 Table; S3 Appendix). We restricted the curated analysis to 5 genomes with more conserved copies, where these are on long contigs/scaffolds.

In the ciliates *Stentor coeruleus* (GCA_001970955.1) *and S*. *roeselii* (GCA_006503475.1), several preserved ORFs were found encoding proteins varying from 381 to 425 aa containing the C-terminal DDE_Tnp_IS1595 domain. However, these proteins show considerable divergence among each other (Fig 2D; S4 Table). We were not able to find conserved TIRs and TSDs around the ORFs and very short intergenic regions separate them from the neighboring ORFs. CENSOR results for these proteins revealed similarity with known *Merlin* elements, although the DDE motif is not conserved in several ones (Figs 2 and 3). Some copies are probably the product of segment duplication rather than transposition since the nearest genes are shared (S.c_G1 and G6), and it is possible to observe syntenic conservation between both species (S.r_RSEH01000003.1 and S.c_G1 copies; S.r_RSEH1000098.1 and S.c_MPUH01000167.1). The absence of TIRs and TSDs indicates these copies are old *Merlin* insertions, and the conservation of transposase suggests they passed through domestication since rapid pseudogenization would be expected for ancestral copies.

In *Perkinsus marinus* (GCF_000006405.1), two *Merlin*-related preserved ORFs and several remnants of *Merlin* were found. The 350-aa proteins differ by 58% while both have conserved DDE_Tnp_IS1595 domain (S4 Table) and DDE motif (Fig 3). It was not possible to identify the TIRs and TSDs, which raises doubts about the activity of these sequences and the reason behind the conservation of transposase.

*Symbiodinium microadriaticum* (GCA_001939145.1) contains several preserved ORFs encoding to *Merlin*-related proteins and several remnants. The proteins vary in size from 181 to 445 aa and contain the DDE_Tnp_IS1595 domain, whereas in some of them the DDE motif is not conserved (Figs 2 and 3). The proteins are divided into two major clusters (Fig 2) and additional subdivisions are possible due to sequence divergence. We could not identify conserved TIRs and TSDs, except for one group of sequences that represent a *bona fide* transposon family, which we call Sy.m_F1. In sequences from groups G4 and G5, we observed the expected 5’-CC-3’ conservation in the 3’ end of the alignment, while the 5’ border could not be identified, hence representing copies in process of degeneration. Sequences from groups G1, G2 and G3 may be remnants of very old insertions and the conservation as ORFs suggests they could have been domesticated. The G2 copies seem to have been amplified by segment duplication rather than transposition.

**Fig 4:**
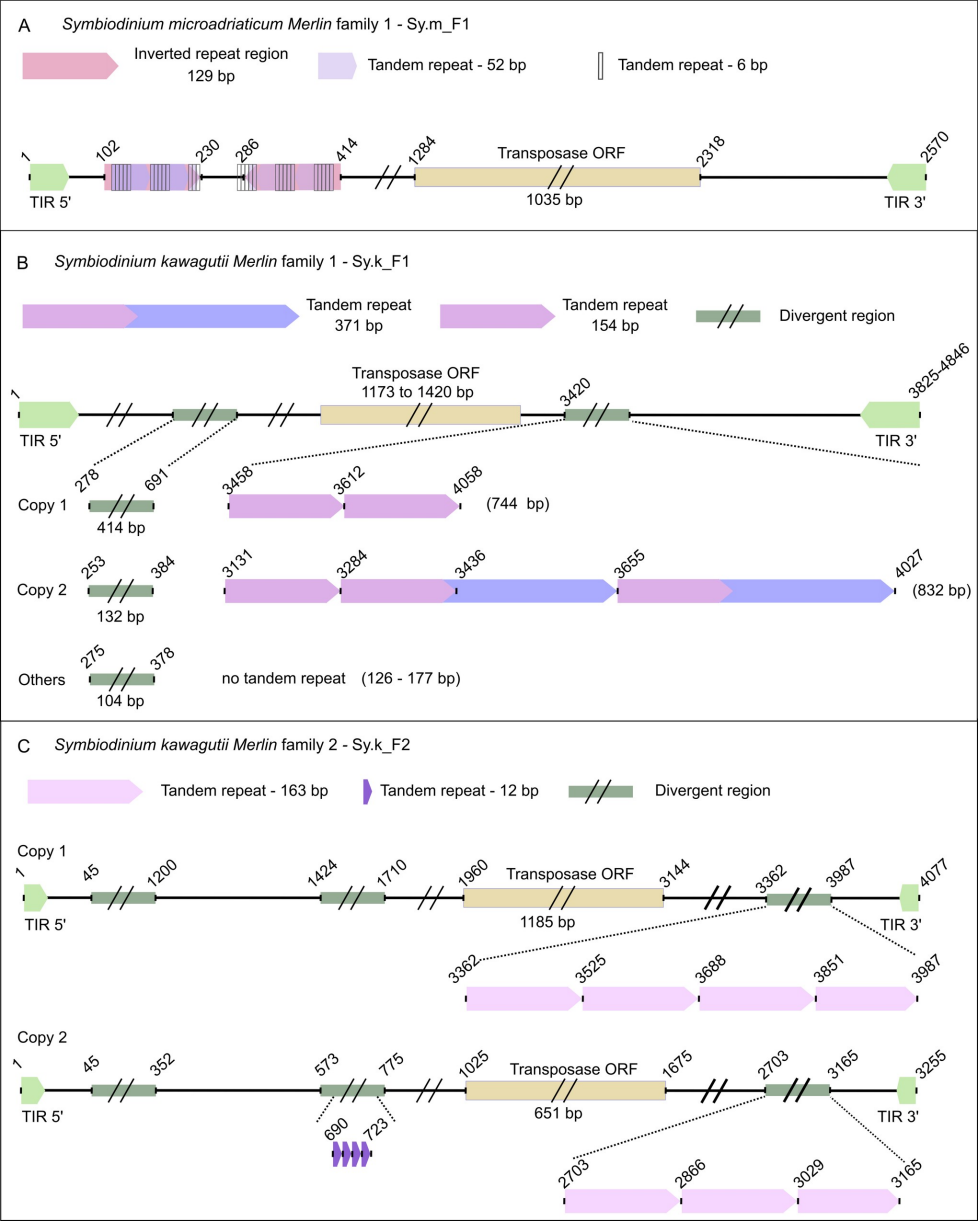
Schematic representation of *Merlin* families containing tandem repeats. A) Representation of Sy.m_F1 from *Sy*. *microadriaticum* containing a complex pattern of repeats in the 5’ region of the element. B) Representation of Sy.k_F1 from *Sy*. *kawagutii* showing a 5’ region that has high divergence among copies in sequence and size and a second divergent region in the 3’ end that contains tandem repeats in two copies. Copy 1—VSDK01027557.1, Copy 2—VSDK01013050.1. C) Sy.k_F2 from *Sy*. *kawagutii* with three indicated divergent regions. Due to missing data, we cannot estimate the size of the first region. The second divergent region contains 4 units of a 12-bp repeat in copy 2, while the third divergent region contains a 163-bp tandem repeat. Copy 1—VSDK01020766.1, Copy 2—VSDK01003368.1.

The copies of Sy.m_F1 present 51-bp TIRs and 8-bp TSDs with no clear consensus sequence (Fig 2). The alignment of most conserved copies shows high similarity within 2.57 kb, indicating very recent insertions. There is a complex pattern of repeats in the 5’ region of the element (Fig 4), including 2.3 units of a 52-bp tandem repeat that contains 5 units of a 6-bp tandem repeat, this region being duplicated in the opposite orientation. CENSOR does not indicate similarity with any known eukaryotic *Merlin* and the DDE_Tnp_IS1595 domain is poorly conserved as suggested by the high e-value (2e-01) (S4 Table), yet the DDE motif is conserved (Fig 3). Blastp shows identity around 30% with *Merlin* from *A*. *algerae* (31% coverage, e-value 3e-04) and with an IS*1595* family from *Taibaiella helva* (46% coverage, e-value 1.1e-02). Although the low similarity with both prokaryotic and eukaryotic transposases and the low conservation of the DDE_Tnp_IS1595 domain, the transposase is seemingly functional since the different composition of TSDs among copies show that they are products of transposition. Thus, this is a divergent but functional and active *Merlin* family.

In *Sy*. *kawagutii* (GCA_009767595.1), we found sequences homologous to *Merlin* that fell into two groups (Fig 2) corresponding to different families. Sy.k_F1 has several copies with high conservation in the coding sequence while the 5’ and 3’ regions are divergent, and some copies contain tandem repeats (Fig 4). The DDE_Tnp_IS1595 domain is predicted with high confidence (S4 Table) and the DDE motif is conserved (Fig 3). Some copies also have a C-terminal zinc finger domain (ZZ superfamily; cl00295). The most intriguing feature of this family is that the 5’ and 3’ TIRs are very divergent, presenting 12 mismatches in the first 26 positions (Fig 2; S1 Fig). We were able to determine the limits of the element and the TSDs via the alignment of several copies, but the internal limits of TIRs remained unclear. Few mismatches between 5’ and 3’ TIRs were found for some *Merlin* families [14], nevertheless, this is the first report of highly imperfect TIRs. The identified copies contain one additional 1-kb ORF overlapping with the transposase in the opposite orientation. The predicted protein has no similarity with sequences available in the NCBI and no domain was predicted, being probably generated by chance and kept in all copies due to the conservation in the transposase gene. The conservation of TSDs indicates that despite the great divergence between 5 and 3’ TIRs, these copies were amplified by transposition and most insertions are relatively recent, this being a seemingly active family. Sy.k_F2 is composed of only 2 copies that have TIRs of 26 bp (Fig 2), and also have conserved and divergent regions (Fig 4). Copy 1 contains 4 units of a 163-bp tandem repeat that is present as 3 units in copy 2, which carries a 12 bp-long tandem repeat. The DDE_Tnp_IS1595 domain and DDE motif (Fig 3) are conserved.

All the new TE families described here have the 5’ TIR that initiates with the nucleotides GG (Fig 2), as shown for all other *Merlin* families [14] and all contain the expected DDE_Tnp_IS1595 domain. Hence, we are confident to considered them as members of the *Merlin* superfamily. Imperfect TIRs were common in the families that we described. This feature is found from several active TEs from different superfamilies and in some cases, TIRs are completely absent [45–47]. It has been shown that for *Tc1/mariner*, the transposase binding has different affinities to imperfect TIRs diminishing the transposition rate [47, 48]. Although we have no evidence that *Merlin* behaves like *Tc1/mariner*, we know that TIR sequences are expected to coevolve with the transposase sequence [14, 49]; thus, a suboptimal arrangement for transposition could be positively selected since high rates of transposition could be detrimental to the host.

### First report of DNA transposon in kinetoplastid protists

From 89 kinetoplastid genomes analyzed (S4 Table), we found *Merlin*-related sequences in the free-living *B*. *saltans* (GCA_001460835.1) [50, 51] and the parasitic *Perkinsela* sp. CCAP 1560/4 (GCA_001235845.1) [52]. Only retrotransposons were previously reported for these [51, 52] and other kinetoplastids, such as members of the human-pathogenic genera *Trypanosoma* and *Leishmania* for which high-quality genomes are available [53–55].

The best tblastn hit for *B*. *saltans* and *Perkinsela* sp. produced a low score (57.8 and 70.9, respectively), showing around 40% identity with the query over a region of 100 aa, but with a significant e-value (1.56e-10 and 8.32e-16, respectively). The presence of the DDE_Tnp_IS1595 domain confirms that this is not a spurious result (S4 Table). However, since we found only a few divergent copies of *Merlin* in both species, the first step was to exclude sequence contamination, the most probable source of which in the case of *B*. *saltans* is the feeder bacteria and for *Perkinsela* sp., it is its host *Paramoeba pemaquidensis* [52]. Still, bacterial contamination could happen at any point along the sequencing process. In this case, blastp searches against the NCBI nr database would possibly show high similarity with some bacterial sequences, as was the case for some other species, yet the blast results revealed only around 30% identity with IS*1595* (S5 Table). Moreover, we did not find *Merlin* in the *P*. *pemaquidensis* genome (GCA_002151225.1). This was expected since the above-discussed genome assemblies were well treated to eliminate possible sequence contaminations [51, 52].

To further verify the presence of *Merlin* in *Perkinsela* sp., PCR was performed using 5 pairs of primers that anneal to the transposon copies and neighboring genes. All 5 combinations of primers presented amplification of expected size, suggesting that the genome assembly is correct and indeed contains *Merlin* (S1 File). Thus, there is strong evidence for *Merlin* being present in these kinetoplastid flagellates. Since this sequence element is clearly absent from related trypanosomatid genomes, it is reasonable to speculate that *Merlin* was present in the last common ancestor of Kinetoplastea predicted to exist about 1 billion years ago [56], and is maintained until present in the basal lineages, while it was lost in the more derived and obligatory parasitic trypanosomatids. The reasons behind the maintenance of this TE in the extremely reduced genome of *Perkinsela* sp. is an intriguing question to be addressed in the future.

### *Merlin* transposase is preserved yet inactive in *Perkinsela* sp. and *B*. *saltans*

**Fig 5:**
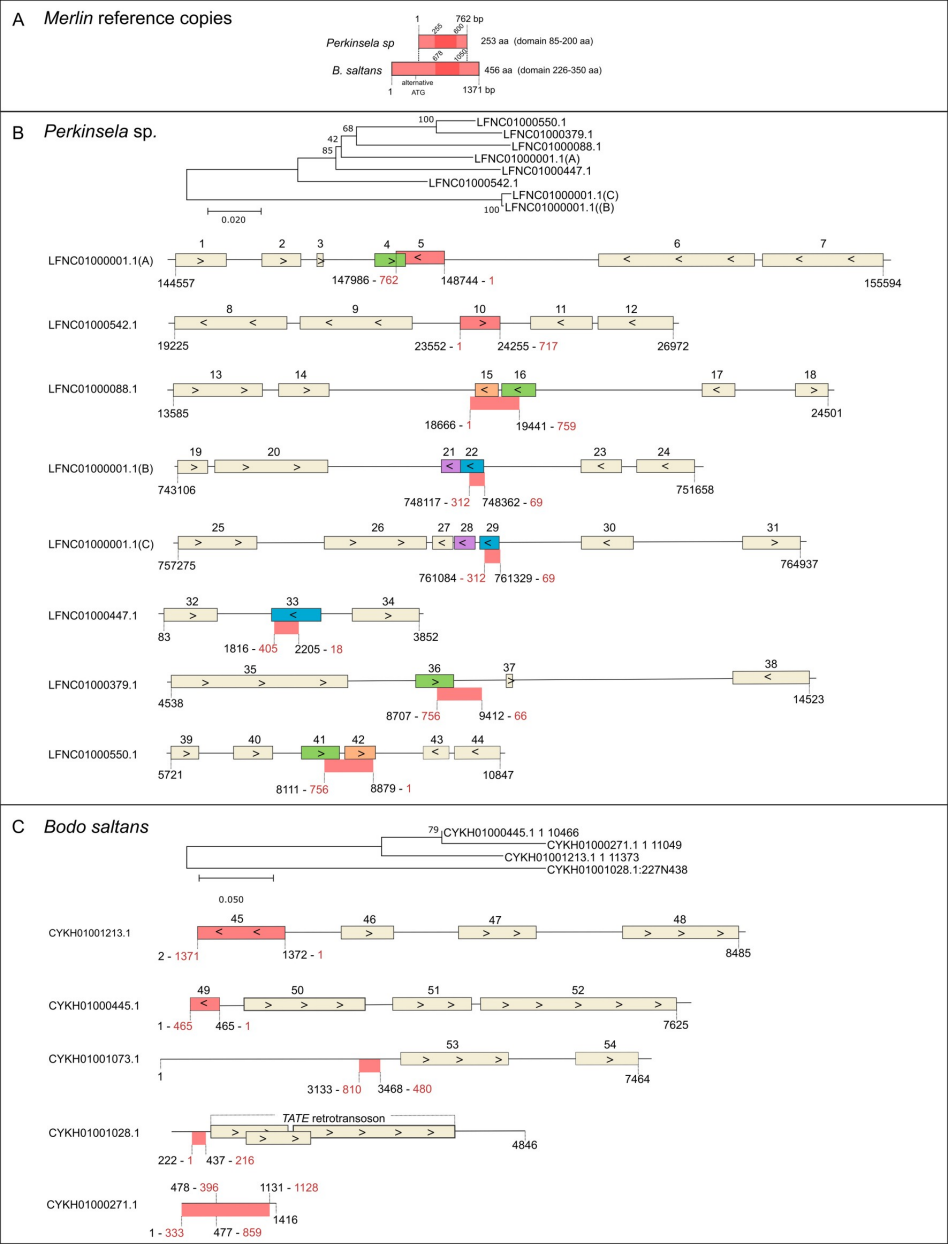
*Merlin* copies from *B*. *saltans* and *Perkinsela* sp. A) Representation of the most conserved *Merlin* copy from each species. An alternative internal ATG is shown. Both proteins possess the DDE_Tnp_IS1595 domain whose coding region is represented by dark color. B) Neighbor-joining tree of *Merlin* copies based on nucleotide sequences and the representation of their genomic context in the *Perkinsela* sp. genome. C) Neighbor-joining tree of *Merlin* copies based on nucleotide sequences and the representation of their genomic context in the *B*. *saltans* genome. ORFs are represented with boxes and numbers and the arrows indicate their direction. Additional information on genes is available in S6 Table. Colored boxes are related ORFs. Red boxes are *Merlin* copies and red boxes without an outline are the non-coding regions with similarity to *Merlin* protein in the tblastn. The relative position of the alignment with *Merlin* reference copy is written in red.

The *Merlin* copies LFNC01000001.1(A) from *Perkinsela* sp. and CYK01001213.1 from *B*. *saltans* are the highest conserved ones in each genome and were used as reference copies (Fig 5A). They are 762 bp and 1.29 kb long in *Perkinsela* sp. and *B*. *saltans*, respectively (although there is an alternative start codon in the latter species). The predicted proteins have 43% identity and 64% similarity in a 219-aa conserved region. The structure of *Merlin* from *Perkinsela* sp., as predicted by Phyre2, indicates similarity to c3hosA, a *Tc1-Mariner Mos1* element from *Drosophila mauritiana* (94.2% confidence, 12% identity) (S2 Fig), while the confidence for *Merlin* from *B*. *saltans* is lower (57.72% confidence). Both proteins retain the characteristic DDE motif (Fig 3), indicating that they still can be functional transposases.

The comparisons of copies in both species revealed low conservation as can be seen by the long branches in the nucleotide sequence trees (Fig 5B and 5C). The *Perkinsela* sp. copies LFNC01000001.1(B) and (C) are possibly derived from segment duplication or assembly artifact.

It was not possible to establish the limits of copies and no conserved TIRs and TSDs were found in *Perkinsela* sp., suggesting *Merlin* is likely very ancient and no longer mobilized. The *Merlin* reference copy (Fig 5B; box 5) is annotated as hypothetical protein XU18_0102 (KNH09417.1) and is located in a strand switch region (SSR), same as some other copies. The *Merlin* ORF found in LFNC01000542.1 (Fig 5B; box 10) is not annotated and is located in the opposite orientation as compared to the neighboring genes, with the predicted protein carrying the DDE_Tnp_IS1595 domain. These copies of *Merlin* in *Perkinsela* sp. possess only 85% identity on the nucleotide level for the entire ORFs alignment and 81% identity on the amino acid level in the conserved core alignment of 199 aa.

Interestingly, we noticed some overlapping ORFs, in both the same and the opposite orientations to *Merlin* copies, some of which are annotated genes (Fig 5B; S6 Table). Boxes 4, 16, 36 and 41 comprise related ORFs that overlap with *Merlin* in the opposite orientation. The ORF 33 encodes a 258-aa protein with no predicted domain which, however, shares similarity in the C-terminal portion with *Merlin*. Considering the similarity on the nucleotide level throughout the entire ORF, this is likely a copy of *Merlin* that underwent frameshift mutations. Moreover, two other short ORFs also seem to have originated from *Merlin* (boxes 15 and 42). These findings are exciting, since these ORFs may represent new genes derived from *Merlin*, although there is currently no evidence regarding their expression.

The only conserved encoding copy in *B*. *saltans* (Fig 5C; box 45) is located at the beginning of contig CYKH01001213.1. It was not possible to analyze the presence of TIRs as no sequence for the 3’ region of the element is available. The same applies to the copy located in contig CYKH01000445.1 and CYKH01000271.1. The other hits correspond to very degenerate copies, generally located in the opposite orientation concerning the neighboring genes. Thus, the current genome assembly of *B*. *saltans* does not allow drawing conclusions regarding its activity, however, we would expect more similar copies in the case of active elements.

The orientation of *Merlin* insertions in both kinetoplastid genomes is an interesting point. Unlike other eukaryotes, genes in kinetoplastids are organized in polycistronic units and the transcription initiation by RNA polymerase II preferentially occurs at divergent SSRs [57, 58]. The TE copies inserted in the same orientation as near genes may be eliminated faster from the genomes since they would be always expressed in the sense strand of the polycistronic transcript. Correspondingly, copies inserted in the opposite orientation or in the SSR region (as is the case for *Merlin* in both species) could be in “safe havens”, reducing their deleterious effects on the host.

We cannot discard the possibility that genome assembly issues impaired us from finding complete copies of *Merlin* in *B*. *saltans* and *Perkinsela* sp. Recently, we have detected complete copies of *VIPER* retrotransposons in two *Trypanosoma cruzi* Dm28c assemblies sequenced by PacBio that were missed in 454-based assemblies [59]. The former technology generates long reads and thus allows better quality assembly of the repetitive sequences, which is not yet available for *B*. *saltans* and *Perkinsela* sp.

In case the *Merlin* copies of *B*. *saltans* and *Perkinsela sp*. are indeed inactive, we wondered why is the transposase gene conserved. Even for inactive copies, one would expect to find TIRs with or without mismatches, yet they are completely absent, indicating an ancient origin of these insertions. It follows that in such case, the transposase would be subject to pseudogenization. Hence, the conservation of *Merlin* transposase could be the result of domestication, a well-documented process in diverse eukaryotes, including kinetoplastids [60, 61].

### *Merlin* and IS*1595* family evolutionary tree

**Fig 6:**
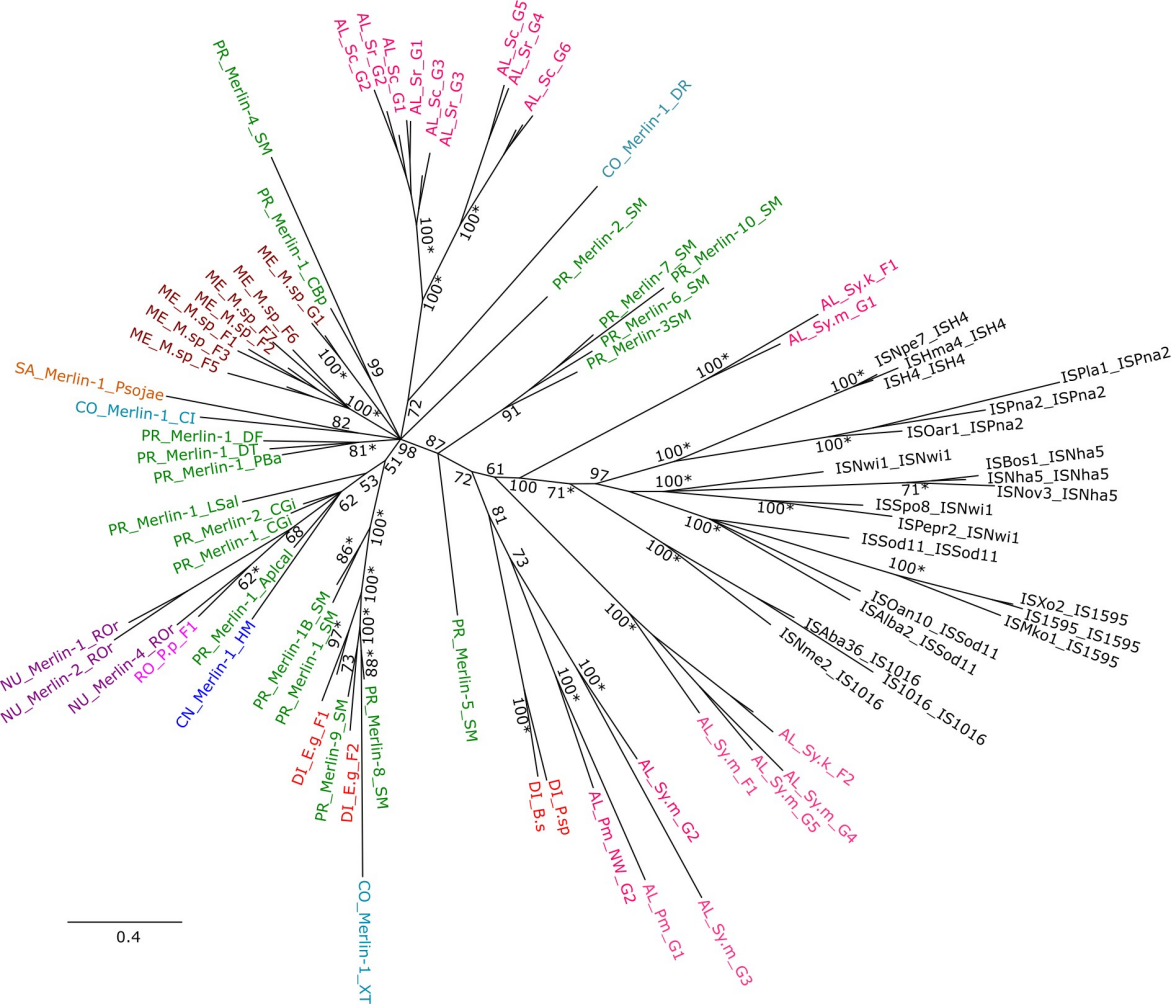
Unrooted 50% majority rule consensus Bayesian tree (WAG + G) of *Merlin* and IS*1595* group sequences based on the amino acid sequence of the conserved transposase domain DDE_Tnp_IS1595 (168 positions). Posterior probability values (PP) are indicated near the nodes and some of the values from derived clades were omitted. The * sign near PP values indicates the clade was supported with bootstrap higher than 50 in the ML tree. *Merlin* sequences from different taxonomic groups are highlighted in different colors and identified with initials: AL–Alveolata, CN–Cnidaria, CO–Chordata, NU–Nucletmycea, PR–Protostomia, SA–Stramenopiles, RO–Rhodophyceae, ME–Metamonada, DI–Discoba. Information of Repbase *Merlin* and IS*1595* sequences are found in S1 and S2 Tables, respectively. *Merlin* sequences characterized in this work are identified by the initial of taxon followed by the abbreviation of species name (S.r–*Stentor roeselii*; S.c–*S*.*coeruleus*; P. p—*Porphyridium purpureum*; Sy.m–*Symbiodinium microadriaticum*; Sy.k–*Symbiodinium kawagutii;* P.m–*Perkinsus marinus*; E.g*–Euglena gracilis;* B.s *-Bodo saltans;* P.sp*–Perkinsela* sp.) and the group or family.

An evolutionary tree of curated *Merlin* families was constructed based on the conserved transposase domain DDE_Tnp_IS1595 (Fig 6) and was presented as unrooted, given the absence of a well-supported outgroup choice, since we assume that eukaryotic *Merlin* could have independent origins. Members from the seven groups of IS*1595* family were included in the tree and formed a monophyletic clade while the internal monophyly of IS*Nwi* and IS*Sod11* were not recovered. We can see that all eukaryotic elements also form a monophyletic group that could suggest a common ancient origin of these sequences.

Sequences from the same taxon such as Protostomia, Alveolata and Chordata, are grouped in distinct clades and most eukaryotic clades are branching from the same node. These observations reinforce the idea of a great divergence time of these sequences with several independent ancient diversification events. No clear cases of horizontal transfer were identified, although it could explain some inconsistencies in the relationships, such as involving *E*. *gracilis* and *P*. *purpureum*. As for other TEs, the stochastic loss is certainly part of the complex evolutionary pattern.

*Merlin* from *B*. *saltans* and *Perkinsela* sp. were grouped in the tree, corroborating the idea that *Merlin* is ancient in kinetoplastids. An acquisition via independent horizontal transfer is a less plausible scenario since it should be from a related source and both species have very distinct ecological niches.

We can observe that the IS*1016* is the closest related IS group to the eukaryotic *Merlin*, corroborating the initial hypothesis [14]. However, considering the greater diversity of *Merlin* sequences that were not included in this tree, we cannot rule out the possibility that other *Merlin* elements would have a closer similarity to other IS*1595* groups. In fact, including a higher number of sequences, leads to a largely unsolved tree (S3 Fig) where most clades are branching from the base, possibly due to insufficient phylogenetic signal reflecting very ancient separation events. The problem of character insufficiency is well known for modest size D-D-D/E-type transposases resulting in poor resolution of most phylogenies [62].

### Final remarks

DNA transposons are important components of eukaryotic genomes with great diversity at the superfamily and family levels. The relatively recently described *Merlin* superfamily seems to be less widely distributed as compared to other superfamilies such as *Tc1-Mariner* and *hAT* [16]. Here, we have described *Merlin-*related sequences for the first time in Alveolata, Rhodophyceae, Metamonada and Discoba. The sequences identified from all taxa, either complete copies, remnants, or derived genes, contain the characteristic domain DDE_Tnp_IS1595, and there is no evidence for recent horizontal transfer from bacterial ISs, thus all sequences were classified as *Merlin* superfamily regardless of having a common origin.

Our results indicate that *Merlin* is more widespread than thought before and its presence in all major eukaryotic supergroups for which complete genomes are available (Amorphea, Diaphoretickes, Metamonada and Discoba) is compatible with the notion that *Merlin* is a truly ancestral genetic element. Feschotte [14] suggested that *Merlin* and the IS*1016* proteins belong to a distinct monophyletic group of transposases that have differentiated from other transposases before the divergence of eukaryotes and prokaryotes. The evolutionary tree obtained with the curated sequences supports this idea. However, due to the diversity of *Merlin* found, and the lack of resolution in the more embracing tree, we cannot rule out the possibility that this superfamily is a result of independent invasions of related prokaryotic IS*1595* family members into the eukaryote genomes. Moreover, the patchy distribution and diversity that we see in some groups are better explained by multiple horizontal transfers of *Merlin*.

The presence of conserved *Merlin*-related sequences with no evidence of mobilization capacity preservation was observed in some species, suggesting that *Merlin* may have been domesticated during evolution. Several studies indicate that transposases were co-opted to act in DNA binding, modulation of chromatin structure and TE repression [9, 63]. They are also associated with programmed genome rearrangement in the ciliates *Paramecium*, *Tetrahymena* and *Oxytricha* [64], and in some cases, the transposase domain and the D-D-D/E motif remain conserved [63]. Thus, we describe several potential *Merlin* domestication cases that worth further investigation.

Concerning the putatively active *Merlin* families, we can highlight that several of them exhibit signs of recent transposition activity. *Merlin* was successful in colonizing and diverging in the *Monocercomonoides* sp. genome where 7 potentially active families were identified. This co-existence of divergent *Merlin* families in the same genome was already reported [14, 17]. We also observed that *Merlin* can have highly imperfect TIRs as seen for Sy.k_F1, a feature not described before for this superfamily. Finally, another interesting finding is the observation of tandem repeats inside *Merlin* sequences. Although this was not described before, we can observe that several of Repbase *Merlin* families also have internal tandem repeats (S1 Table). The close relationship of tandem repeats and TEs has been recently well documented, with several micro, mini and satellite DNAs found embedded within TEs [65–70]. The existence of tandem repeats in multiple families and copies of *Merlin* indicates this TE could help to spread tandem repeats by transposition as proto-satellites that could be next amplified and homogenized such as the model suggested by Paço and colleagues [67].

Importantly, we document for the first time DNA transposons in the kinetoplastid genomes that were thought to be devoid of these genetic elements. The absence of TIRs and TSDs in the *Merlin* copies found in *Perkinsela* sp. contrasts with the conservation of the transposase coding region. Thus, it is possible that complete copies were not assembled, or the transposase is being maintained for a currently unknown cellular function. Our findings represent a starting point for understanding the impact of these sequences on protists and reveal greater diversity of TEs than thought previously.

## Supporting information

S1 Fig Alignment of flanking regions of most conserved copies of *Merlin* family 1 from *Sy*. *kawagutii* (Sy.k_F1). The first and last (reverse complement) 130 nucleotides of copies were aligned and it is possible to observe highly imperfect TIRs with no clear limit. TSDs for each copy are shown highlighted in different colors.
(TIF)

S2 Fig Secondary structure prediction of *Merlin* reference protein from *Perkinsela* sp. modeled used Phyre2. The secondary structure was predicted using *c3hosA* template, a *Tc1-Mariner Mos1* element from *Drosophila mauritiana*. The structure was predicted with 94.2% confidence.
(TIF)

S3 Fig Bayesian tree (LG + G) of *Merlin* and IS*1595* group sequences. The tree is based on the amino acid sequence of the conserved transposase domain DDE_Tnp_IS1595 (142 positions) and was rooted by the midpoint.
(PNG)

S1 File Information on PCR for confirming the presence of *Merlin* elements in *Perkinsela* sp. (PDF)

S2 File Alignment of the conserved transposase domain DDE_Tnp_IS1595 of curated *Merlin* sequences and IS*1595* used to generate the tree shown in Fig 6. (ALN)

S3 File Alignment of the conserved transposase domain DDE_Tnp_IS1595 of *Merlin*/IS*1595* used to generate the tree shown in S3 Fig. (ALN)

S1 Appendix *Merlin*-related proteins identified by blastp. Sequences are provided as fasta files for each major taxonomic group and the results of the CD-search are also provided.
(ZIP)

S2 Appendix *Merlin*-related sequences identified by tblastn from Rhizaria, Streptophyta, Amoebozoa, Chlorophyta, Hemichordata, Ctenophora and Xenacoelomorpha. Contigs containing positive hits and protein sequences are provided as fasta files. The results of the CD-search are also provided. A README file is provided with additional information about files.
(ZIP)

S3 Appendix Sequences analyzed in this work from Discoba, Metamonada, Rodophyceae and Alveolata. Contigs containing positive hits for each species are provided as fasta files. Nucleotide sequences from each *Merlin* group or family are also provided. All protein sequences were provided in a single file. A README file is provided with additional information about files.
(ZIP)

S1 Table Summary of main features of *Merlin* canonical families available in the Repbase23.11. (XLSX)

S2 Table Information of IS*1595* group sequences used in the phylogenetic tree. (XLSX)

S3 Table Summary of blastp and tblastn against the major taxonomic groups. (XLSX)

S4 Table General results obtained by Rhodophyceae, Rhizaria, Metamonada, Discoba and Alveolata groups. The data is provided in different tabs, including the genomes analyzed, CD-search and CENSOR results and protein divergence. The description of each tab is available in the first tab.
(XLSX)

S5 Table Summary of blastp result against protein nr database using *Merlin* protein sequence from *Bodo saltans* and *Perkinsela* sp. as a query. (XLSX)

S6 Table Information about the ORFs overlapping and near *Merlin* copies that are represented in Fig 5. (XLSX)

S1 Raw images (PDF)
