# Supplementary figures and images for: Distribution of Merlin in eukaryotes and first report of DNA transposons in kinetoplastid protists
Source: PLoS One. 2021 May 6;16(5):e0251133. doi: 10.1371/journal.pone.0251133 (PMC8101967; doi:10.1371/journal.pone.0251133)

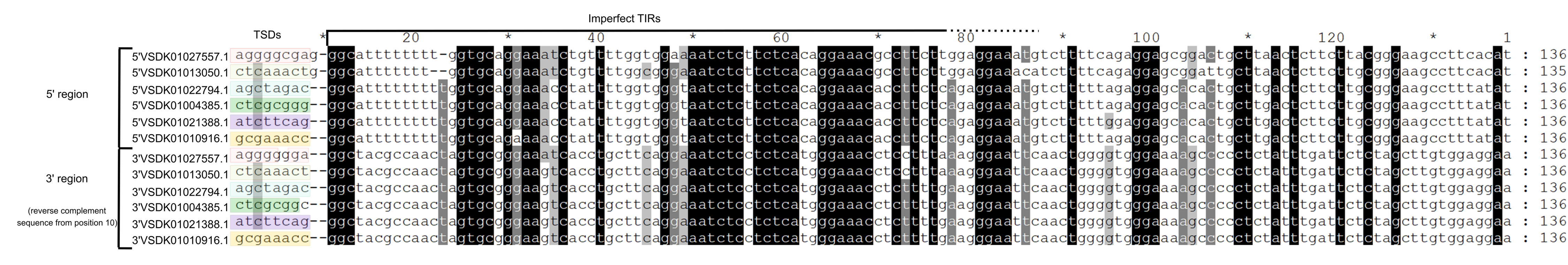

Supplement: S1 Fig — The first and last (reverse complement) 130 nucleotides of copies were aligned and it is possible to observe highly imperfect TIRs with no clear limit. TSDs for each copy are shown highlighted in different colors. (TIF) [file pone.0251133.s001.tif]

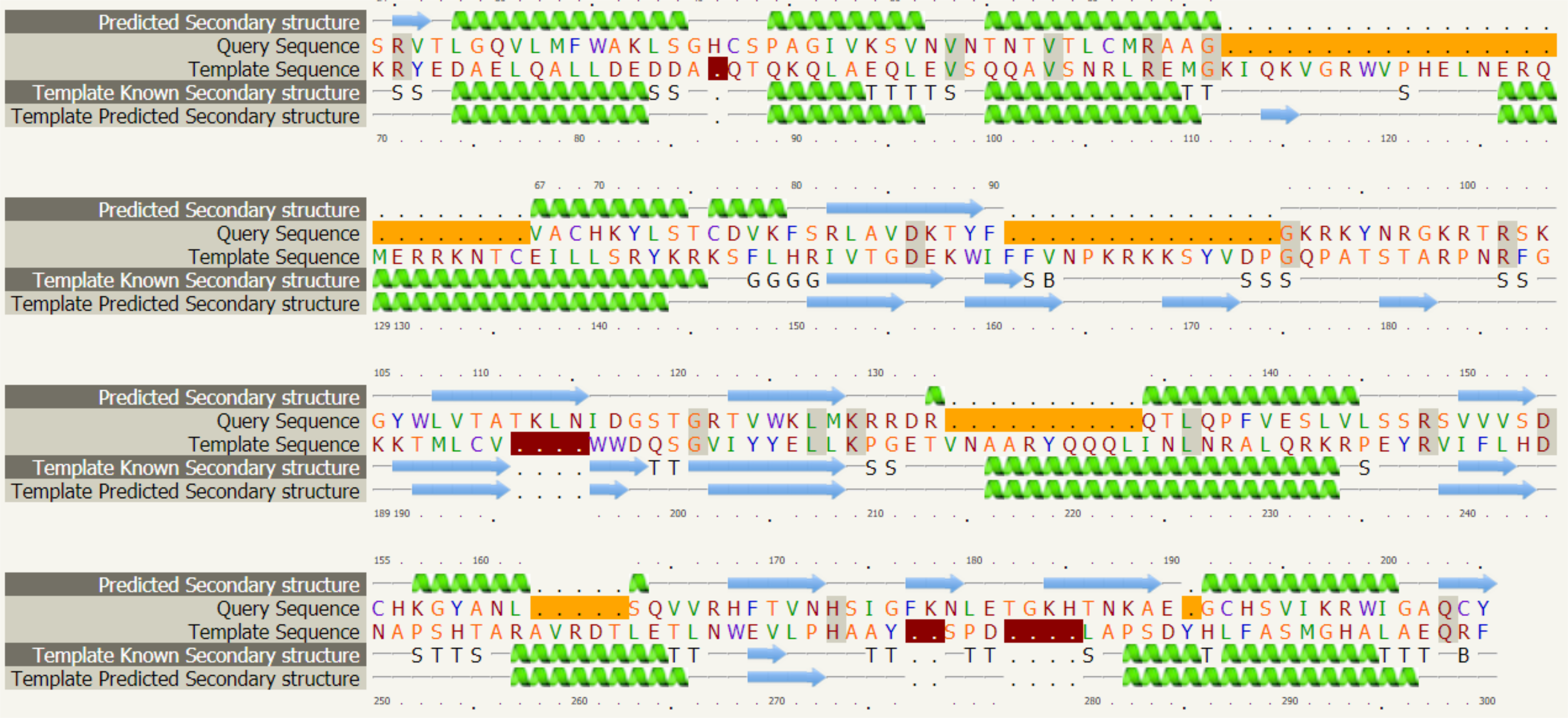

Supplement: S2 Fig — The secondary structure was predicted using c3hosA template, a Tc1-Mariner Mos1 element from Drosophila mauritiana. The structure was predicted with 94.2% confidence. (TIF) [file pone.0251133.s002.tif]

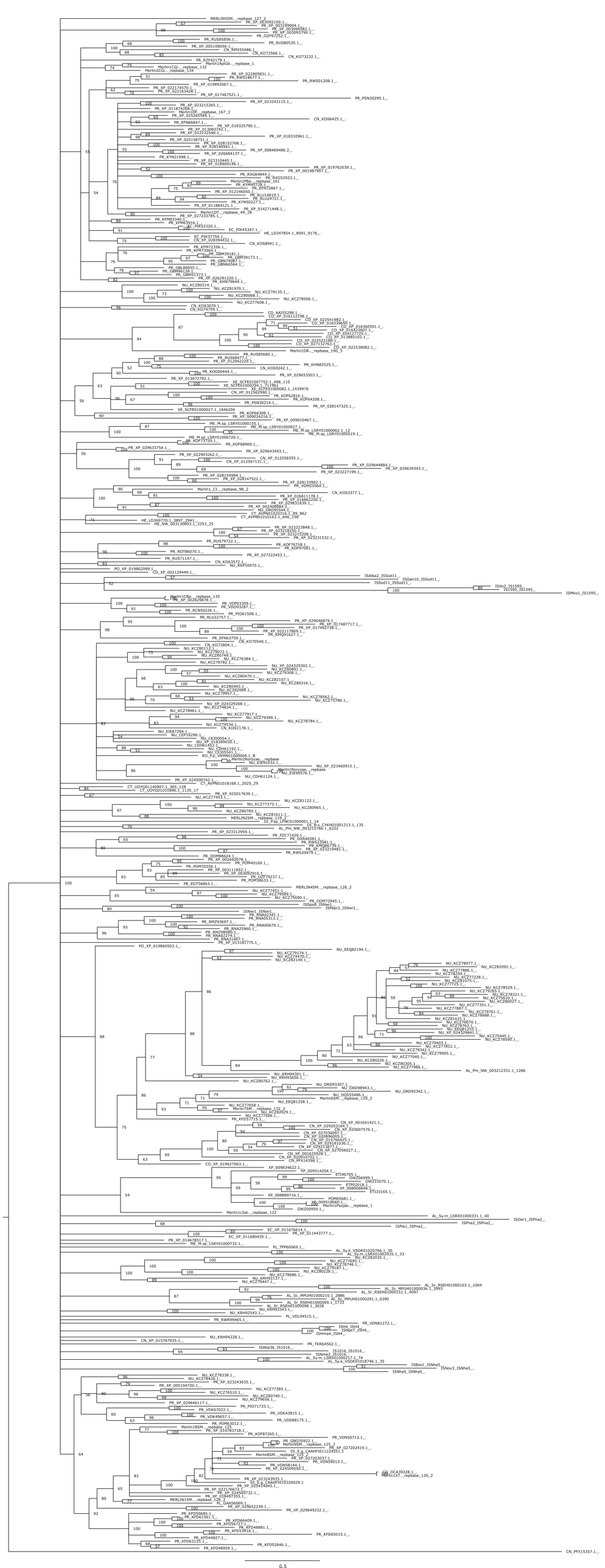

Supplement: S3 Fig — The tree is based on the amino acid sequence of the conserved transposase domain DDE_Tnp_IS1595 (142 positions) and was rooted by the midpoint. (PNG) [file pone.0251133.s003.png]
