# Supplementary material for: Distribution of Merlin in eukaryotes and first report of DNA transposons in kinetoplastid protists
Source: PLoS One. 2021 May 6;16(5):e0251133. doi: 10.1371/journal.pone.0251133 (PMC8101967; doi:10.1371/journal.pone.0251133)
Supplement: S1 File — (PDF) [file pone.0251133.s004.pdf]

Information on PCR for confirming the presence of *Merlin* elements in *Perkinsela* sp.

The DNA of *Perkinsela* sp. (strain CCAP1560/4) was submitted to gradient PCR analysis. The reaction was composed by 12.5 µl of Q5 Hot Start High-Fidelity 2X Master Mix, 1.25 µl of each primer (5 µM) forward and reverse, DNA sample and water until a final volume of 25 µl. Cycling conditions were as following: 98ºC for 2 min, followed by 16 cycles of 98ºC for 20s, 30s at an initial temperature of 65°C, decreasing 1°C per cycle, and 2 min extension at 72°C, more 20 cycles were performed with 98ºC for 20s, 50ºC for 30s, and 72°C for 2 min, with a final extension of 2 min at 72°C.

Primers for three copies of *Merlin* were designed (see table below) to anneal one in the transposon and the other in the next gene. Figure 1 shows the amplification of fragments of expected sizes, showing that the *Merlin* element discarding the hypothesis of to be an artifact of genome assembly.

Table 1: Information about the primers used to confirm the *Merlin* presence in *Perkinsela sp.*

Merlin copies	Near gene	Information of gene	Primers	Expected amplification size (bp)
**Copy 1 LFNC01000001.1: 147986-148744**	Up: *perosamine synthetase* gene - LFNC01000001.1: 151154-153586	single copy, shared with other kinetoplastids, same direction	copy1UpF TAAACTCAGGCTGCCCAAGG copy1R CACGCGTGAAGTGACTAAGC	2572
**Copy 1 LFNC01000001.1: 147986-148744)**	Down: endonuclease III - LFNC01000001.1: 144539- 145330	single copy, shared with other kinetoplastids, opposite direction	copy1DownF GCACATACGATCATGCGCC copy1DownR GTCCTTCTGTGCGCAAATCC	2733
**Copy 2 LFNC01000542.1: 23598 - 24221**	Up: phosphoglycerate kinase LFNC01000542.1: 19228 - 20889	single copy, shared with other kinetoplastids, opposite direction	copy2UpF GCACGCATAAAACGCGTAGG Copy2UpR CACTTTGCACGATCTCTGCG	2880
**Copy 2 LFNC01000542.1: 23598 - 24221**	Down: ring finger protein LFNC01000542.1: 27197-28429	single copy, shared with other kinetoplastids, opposite direction	copy2DownF TCCAGTGCGTCAATCATGGC copy2DownR TCAAGTCCAATTCAAAGTGCCC	3044
**Copy 3 LFNC01000088.1: 18666-19441**	Up: HMG box containing transcription factor TCF LFNC01000088.1: 15241-16029	Multiple copies, Specific from *Perskinsela sp*., opposite direction	copy3UpF GTTCTGCTGACGCAACTTCC copy3UpR CTTTTTCTGCAGCTCTGGCG.	2881

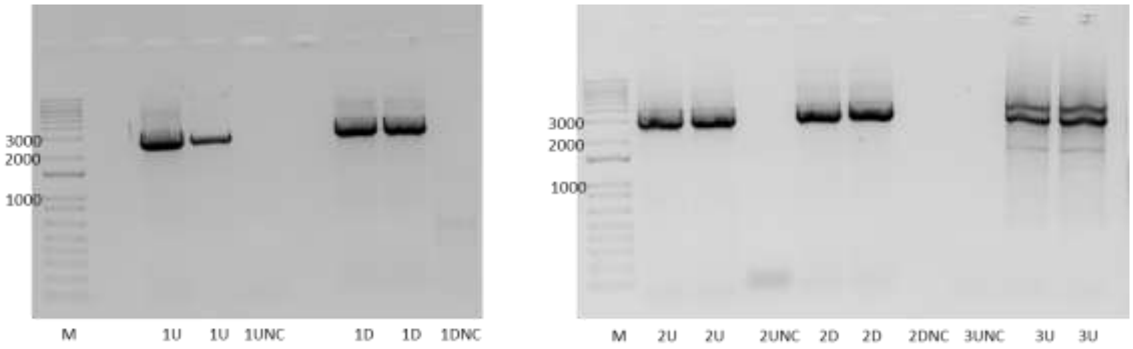

Figure 1: PCR results for the confirmation of *Merlin* element insertion in *Perkinsela* sp. M- 1Kb Plus DNA Ladder; 1D- Copy1GeneDown (expected size: 2771 bp); 1U- Copy1GeneUp (expected size: 2572 bp); 2D- Copy2GeneDown (expected size: 3044 bp); 2U- Copy2GeneUp (expected size: 2880 bp); 3U- Copy3GeneUp (expected size: 2881 bp); 1DNC- Copy1GeneDown negative control; 1UNC- Copy1GeneUp negative control; 2DNC- Copy2GeneDown negative control; 2UNC- Copy2GeneUp negative control; 3UNC-Copy3GeneUp negative control.
