# Supplementary material for: Distribution of Merlin in eukaryotes and first report of DNA transposons in kinetoplastid protists
Source: PLoS One. 2021 May 6;16(5):e0251133. doi: 10.1371/journal.pone.0251133 (PMC8101967; doi:10.1371/journal.pone.0251133)
Supplement: S1 Raw images — (PDF) [file pone.0251133.s016.pdf]

PCR results for *Perkinserla* sp.

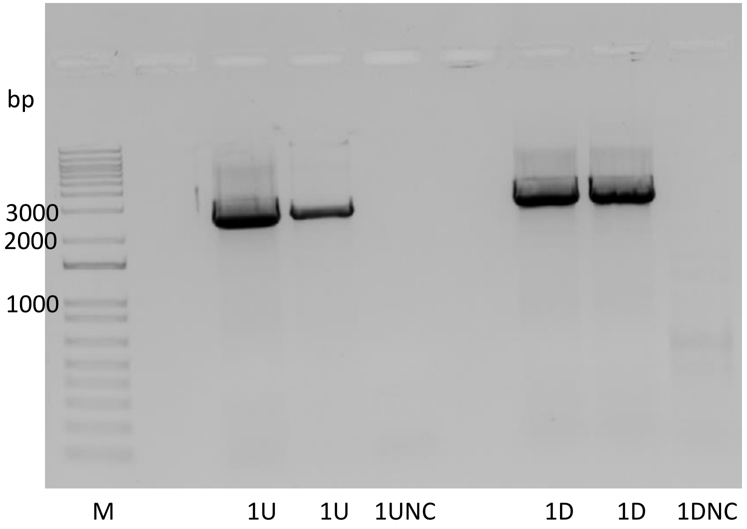

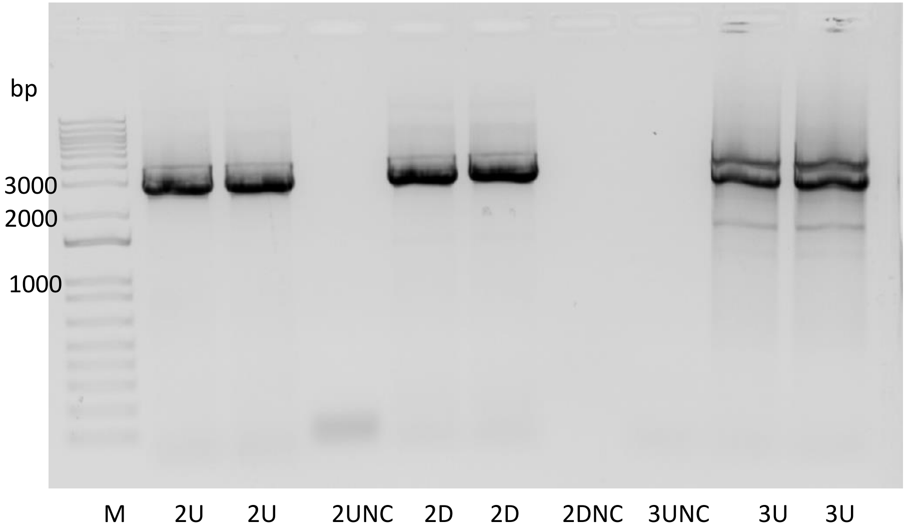

M- 1Kb Plus DNA Ladder
1D- Copy1GeneDown (2771 bp)
1U- Copy1GeneUp (2572 bp)
2D- Copy2GeneDown (3044 bp)
2U- Copy2GeneUp (2880 bp)
3U- Copy3GeneUp (2881 bp)
1DNC- Copy1GeneDown negative control
1UNC- Copy1GeneUp negative control
2DNC- Copy2GeneDown negative control
2UNC- Copy2GeneUp negative control
3UNC-Copy3GeneUp negative control
